# Organizing substitution of oncological follow-up to primary care: perspectives from secondary care providers

**DOI:** 10.1007/s11764-025-01764-x

**Published:** 2025-03-06

**Authors:** Geertje B. Liemburg, Joke C. Korevaar, Annette J. Berendsen, Marjolein Y. Berger, Daan Brandenbarg

**Affiliations:** 1 Department of Primary and Long-Term Care, University Medical Center Groningen, University of Groningen, P.O. Box 196, Groningen, 9700 AD the Netherlands; 2 NIVEL Netherlands Institute for Health Services Research, Utrecht, the Netherlands; 3 Faculty of Health, Nutrition and Sport, The Hague University of Applied Sciences, The Hague, the Netherlands

**Keywords:** Follow-up, Breast cancer, Colorectal cancer, General practitioner, Shared care, Qualitative research

## Abstract

**Purpose:**

The increasing number of cancer survivors has heightened demands on hospital-based follow-up care resources. To address this, involving general practitioners (GPs) in oncological follow-up is proposed. This study explores secondary care providers’ views on integrating GPs into follow-up care for curatively treated breast and colorectal cancer survivors.

**Methods:**

A qualitative exploratory study was conducted using semi-structured interviews with Dutch medical specialists and nurse practitioners. Interviews were recorded, transcribed verbatim, and analyzed using thematic analysis by two independent researchers.

**Results:**

Fifteen medical specialists and nine nurse practitioners participated. They identified barriers such as re-referral delays, inexperience to perform structured follow-up, and worries about the lack of oncological knowledge among GPs. Benefits included the GPs’ accessibility and their contextual knowledge. For future organization, they emphasized the need for hospital logistics changes, formal GP training, sufficient case-load, proper staffing, remuneration, and time allocation. They suggested that formal GP involvement should initially be implemented for frail older patients and for prevalent cancer types.

**Conclusions:**

The interviewed Dutch secondary care providers generally supported formal involvement of primary care in cancer follow-up. A well-organized shared-care model with defined roles and clear coordination, supported by individual patients, was considered essential. This approach requires logistics adaptation, resources, and training for GPs.

**Implications for cancer survivors:**

Integrating oncological follow-up into routine primary care through a shared-care model may lead to personalized, effective, and efficient care for survivors because of their long-term relationships with GPs.

**Supplementary Information:**

The online version contains supplementary material available at 10.1007/s11764-025-01764-x.

## Introduction

The number of cancer survivors has increased in the past decades and is expected to increase even further. This is largely due to an increase of diagnoses in an ageing population and better survival [1]. Cancer survivors usually receive hospital-based follow-up care after treatment. This lasts generally 5 years for patients treated with curative intent and is mainly focused on recurrence detection and monitoring of side- and long-term effects of treatment [2, 3]. The increase of survivors puts pressure on the organization and resources for this type of care. This results in a decades-long debate about the most patient-friendly and efficient organization of follow-up care. However, the literature showed no consensus on the optimal approach of substitution of care to primary care [4–6]. In the Netherlands, general practitioners (GPs) act as gatekeepers to secondary care, referring patients to specialized medical care in hospitals.

GPs could provide valuable advantages in survivorship care by leveraging their knowledge of patients’ medical histories and social contexts for personalized support [7, 8]. Their integration into follow-up care may improve continuity of care and could create the circumstances to offer more tailored follow-up care close to patients’ homes, potentially improving quality of life and patient satisfaction [9]. Several authorities, including The Dutch College of General Practitioners (NHG), have indicated that GPs could play a more formal role in cancer follow-up, as they can provide continuity of care and comprehensive care. No conditions were formulated to guarantee the quality of follow-up care provided by GPs for the most prevalent cancers yet [10].

Studies comparing primary care-led follow-up of cancer patients (solid tumors, including breast and colon cancer) with hospital follow-up reported no differences in recurrence rates and timing of diagnosis, survival rates, and patient satisfaction. This indicates that primary care-led follow-up is safe for a number of most prevalent cancers [4, 6]. Another frequently suggested model of follow-up care is shared care, in which primary and secondary care collaborate, combining the expertise of cancer-specific care with the continuity of care provided by the general practitioner [5, 11, 12]. Despite qualitative evidence on feasibility, there is no agreement on the most appropriate format of formal GP involvement in oncological follow-up, due to differences in healthcare systems [13]. To date, only a few qualitative studies reported opinions of secondary care providers [14–16]. These studies showed that, according to oncologists, GPs could be more formally involved, provided it was in a shared-care setting with clearly defined responsibilities between GP practices and hospitals. We previously investigated the views of GPs and patients on how to realize substitution of care [17, 18]. The views of secondary care providers, in particular, deserve more attention, as they play an important role in current follow-up care and will continue to be crucial in the future, especially in the context of potential shared-care solutions. Therefore, we performed a qualitative study among secondary care providers about how oncological follow-up care for curatively treated breast and colorectal cancer survivors can be structured to formally involve primary care.

## Material and methods

### Study design

A qualitative explorative study was performed using semi-structured individual interviews to allow for in-depth questions regarding possible solutions for the structuring of oncological follow-up care to formally integrate primary care.

### Study population

Dutch secondary care providers (medical oncologists, oncology surgeons, radiotherapists, and gastro-enterologists) and hospital nurse practitioners involved in the follow-up care of breast and/or colorectal cancer were invited to participate. Breast and colorectal cancer are amongst the five most common cancers in the Netherlands [19]. Follow-up appointments for colorectal cancer are initially four times a year, consisting of physical examination, blood tests, radiographic examination, and colonoscopy. Breast cancer follow-up is performed at least once a year and mainly consists of physical examination and mammography [20, 21].

### Recruitment

We sent an invitation letter to care providers in ten hospitals in the Northern and Middle parts of the Netherlands. From the secondary care providers that responded to our invitation, we purposively selected participants with the goal of achieving maximum variation on age, gender, medical specialism, type of hospital (academic teaching hospital or general hospital), and location. First, we selected with the aim to obtain variety in terms of medical specialism (e.g., internal medicine, surgery, gastroenterology, radiotherapy), type of hospital, and location in the Netherlands. We then sampled iteratively within these criteria on variation in age and gender, ensuring a balance between young and old, as well as male and female participants.

### Data collection

Individual semi-structured interviews were held. Based on the literature and (clinical) expertise of the research group, a topic list/interview guide was developed and pilot-tested among two medical specialists. The topic list was adjusted when new topics arose during interviews or data analysis, i.e., an iterative approach was used.

All interviews took place in the hospital where the participants worked and were conducted by either DB or GBL. DB is a clinical epidemiologist with a PhD in primary care cancer research. GBL is an MD who was a GP trainee and a PhD student at the time of the study. Both have received training in qualitative methods and have ample experience with conducting semi-structured interviews. The interviews were audio recorded and transcribed verbatim (by LO and FT) and pseudonymized. Transcripts were checked for inaccuracies by listening to the audio data (GBL). Participants completed a short questionnaire with their demographic data and whether they followed national guidelines. The study is described according to the consolidated criteria for reporting qualitative research checklist (COREQ), as shown in Online Resource 1 [22].

### Data analysis

Thematic analysis with a hybrid approach of inductive and deductive coding was used [23, 24].

The initial coding framework was informed by prior research, including our own previous studies and existing literature on this topic. This guided the development of broad, a priori categories—perceived benefits, perceived barriers, and necessary requirements for primary care involvement—providing a structured starting point for the analysis. Within these predefined categories, we applied an inductive approach to coding, allowing for the identification of new themes and sub-themes emerging from the data (process of open, axial, and selective coding). Two independent researchers (GBL and WvZ) identified codes and themes and possible discrepancies were discussed with a third independent researcher (DB). This analysis was repeated until data saturation, i.e., until no new themes emerged in four consecutive transcripts [25]. Regular meetings with the research group were organized (GBL, DB, JCK, AJB, and MYB) in which the progress and results were discussed by using coding trees to visualize the data. A member check was performed by sending a brief summary to five randomly selected participants to enhance internal validity. Transcripts were coded using ATLAS.ti version 8.4 (GmbH, Berlin, Germany).

## Results

### Participants

Of the 75 invited secondary care providers, 31 care providers responded that they were willing to participate (response rate 41%). Commonly cited reasons for non-participation included being too busy, receiving numerous requests to participate in scientific studies, and the fact that a colleague was already participating. Eventually, 24 secondary care providers from 7 hospitals participated in individual interviews. Data saturation was assumed after the analysis of 20 transcripts, and this was confirmed in the four consecutive interviews.

The study population consisted of 11 males and 13 females with a median age of 49.5 (range 32–64) years. Participants were medical specialists (*n* = 15) and nurse practitioners (*n* = 9) (hereinafter, all are referred to as secondary care providers). Half of the participants (*n* = 12) worked in a surgical department, 10 in internal medicine (including breast care and gastroenterology), and two in radiology. Nine participants were involved in both breast- and colorectal cancer follow-up care, 8 in breast cancer only, and 7 in colorectal cancer only (Table 1). Most medical specialists worked according to the national guidelines; however, reasons to deviate from guidelines were mostly in case of patient-related individual factors like age or personal wishes of the patients (Box 1).

**Table 1:** Participants’ characteristics

ID	Gender	Age (years)	Experience (years)	Work (%)	Type of hospital	Department	Medical specialism	Involvement in breast or colorectal cancer
S01	Female	59	21	80	General	Internal medicine	Medical specialist	Both
S02	Female	39	14	80	General	Surgery	Nurse practitioner	Breast
S03	Male	64	33	100	Academic	Surgery	Medical specialist	Breast
S04	Female	38	4	100	General	Surgery	Medical specialist	Both
S05	Female	49	13	100	Academic	Internal medicine	Medical specialist	Both
S06	Male	38	3	80	Academic	Surgery	Medical specialist	Colorectal
S07	Female	55	32	100	Academic	Internal medicine	Nurse practitioner	Both
S08	Male	54	16	90	General	Internal medicine	Medical specialist	Both
S09	Female	50	30	77	General	Gastroenterology	Nurse practitioner	Colorectal
S10	Male	53	17	90	General	Internal medicine	Medical specialist	Both
S11	Male	38	2	80	Teaching	Surgery	Medical specialist	Colorectal
S12	Female	43	11	100	Teaching	Surgery	Medical specialist	Both
S13	Female	52	6	100	Academic	Surgery	Nurse practitioner	Breast
S14	Female	57	3	100	Teaching	Internal medicine	Nurse practitioner	Breast
S15	Male	62	24	100	General	Surgery	Medical specialist	Both
S16	Female	55	26	70	General	Breast Care Clinic	Nurse practitioner	Breast
S17	Male	41	6	100	General	Surgery	Medical specialist	Colorectal
S18	Male	54	19	100	Teaching	Surgery	Medical specialist	Colorectal
S19	Female	32	8	78	Teaching	Breast Care Clinic	Nurse practitioner	Breast
S20	Male	42	9	100	Academic	Surgery	Medical specialist	Colorectal
S21	Female	51	33	80	General	Surgery	Nurse practitioner	Colorectal
S22	Male	45	19	100	Academic	Radiotherapy	Medical specialist	Breast
S23	Female	39	7	89	General	Breast Care Clinic	Nurse practitioner	Breast
S24	Male	47	12	100	Teaching	Radiotherapy	Medical specialist	Both

The interviews took place in the period between September 2019 and March 2020 and lasted between 24 and 36 min.

**Box 1** Topic list used to guide semi-structured interviews.

**Table:** 

Concerning the follow-up care provided to patients curatively treated for breast cancer (BC) and colorectal cancer (CRC), the following questions were asked:
o What do medical specialist think about the feasibility of (partially) follow-up in primary care o What are the perceived benefits? o What are the perceived barriers? o Which follow-up tests can GPs perform? o What are the requirements for effective care substitution? o What would GPs need to be able to perform (parts of) follow-up care? o For which patients groups could GPs (partially) coordinate follow-up care?
If oncological follow-up is to be (partially) introduced in primary care:
o How should implementation be structured? o Who is going to do what? Who is going to coordinate? o Which stakeholders are involved? o What is necessary for successful cooperation between secondary and primary care?

### Themes

**Fig. 1:**
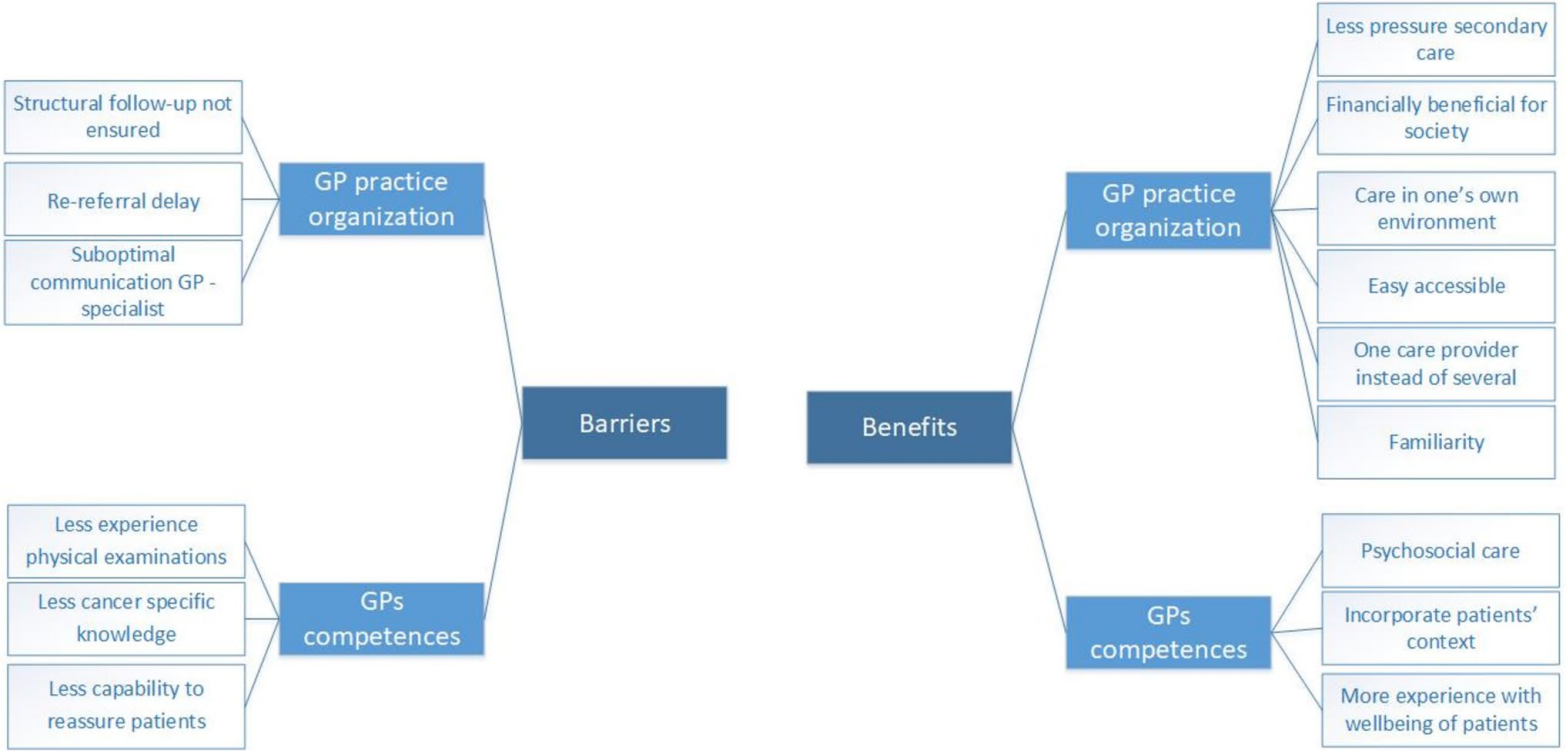
Participants’ views on the benefits and barriers associated with care substitution

**Fig. 2:**
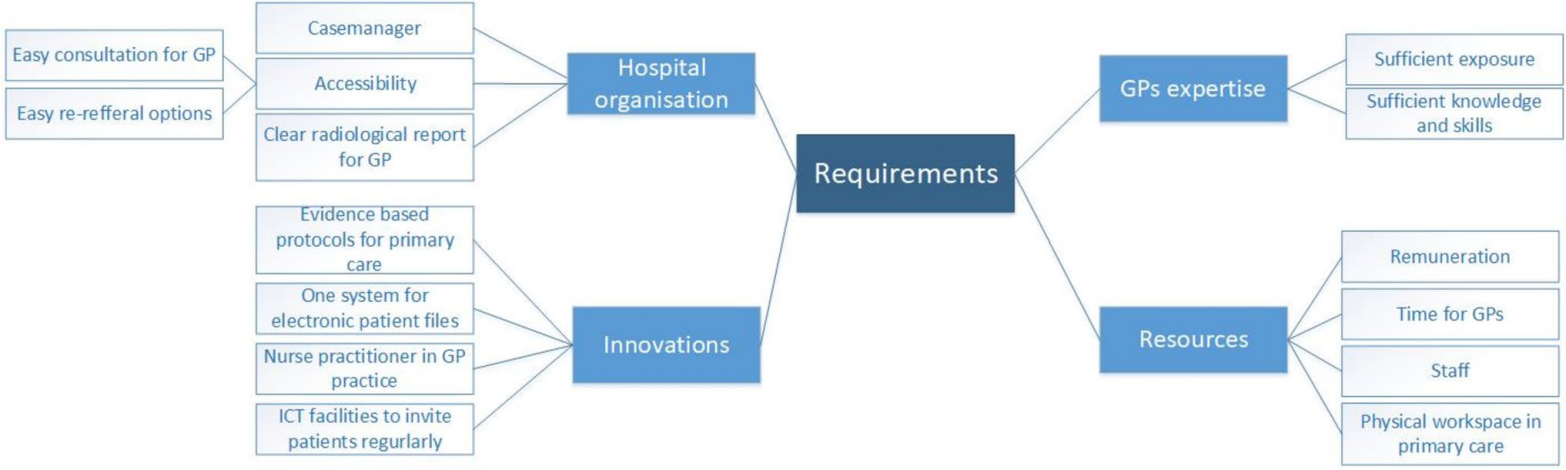
Perceived requirements for care substitution

**Fig. 3:**
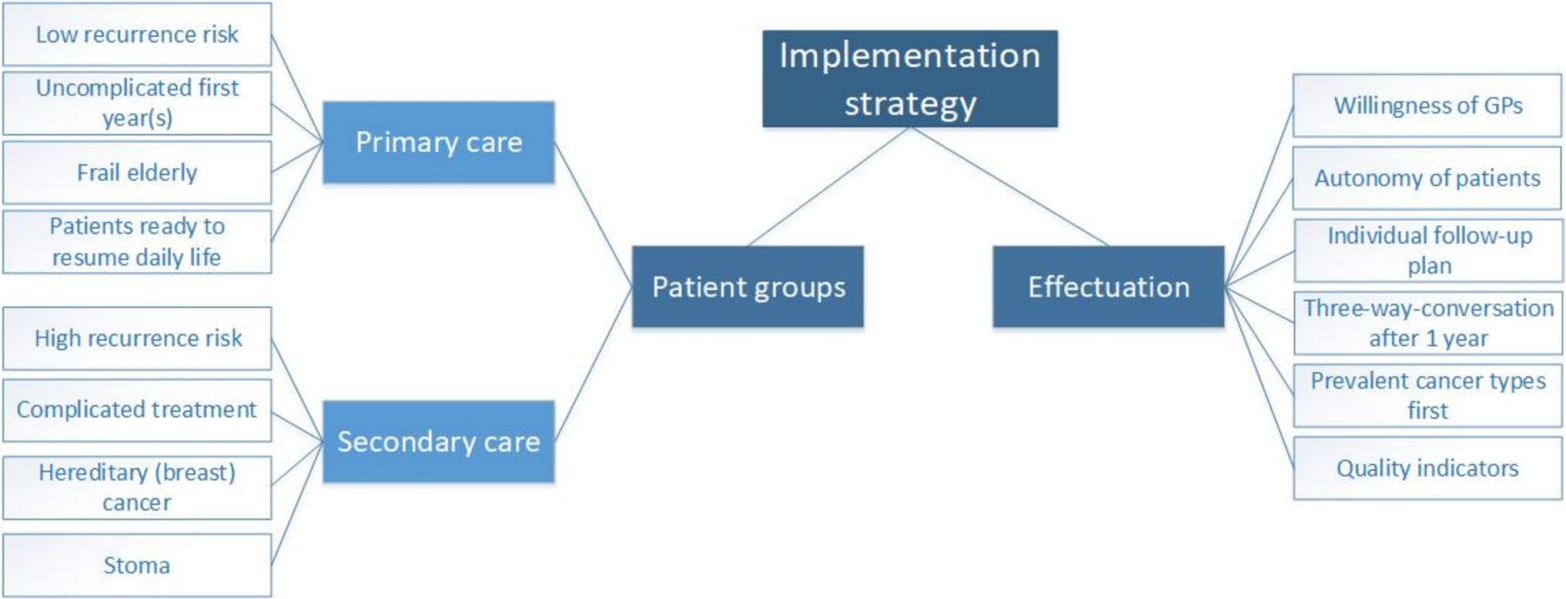
Opinions on implementation strategy including distinguishable groups of patients and approach

Three main themes were identified: “benefits and barriers in GP practice,” “Requirements,” and “Implementation strategy” (Figs. 1, 2, and 3).

### Benefits and barriers in GP practice

Both benefits and barriers, as identified by secondary care providers, emerged from the thematic analysis, with subthemes “GP practice organization” and “GPs’ competences.”

#### GP practice organization

Secondary care providers mentioned several benefits about GP-led follow-up: the GP provides care in patients’ own environment and is easily accessible and trusted. Most of the interviewed surgeons preferred to have one familiar care provider for the patient, such as the GP, rather than several follow-up visits to internists, surgeons, nurse practitioners, and sometimes even a radiotherapist.

The majority believed that GP involvement would indeed contribute to alleviating the increased demand for secondary care. In addition, they thought it would be financially beneficial for society by lowering medical costs and also for patients because mandatory deductibles would decrease. Most internists considered financial reasons to be of minor importance, believing that even if current follow-up care were discontinued, there would still be enough work to ensure that this would not have significant financial consequences for them.

Most medical specialists feared that GPs would not be able to provide structural follow-up care in accordance with the guidelines because GP practices are set up for short episodes of illnesses rather than recurring appointments. Besides, they feared re-referral delays as the GP is located outside the hospital and communication between GPs and specialists is often complicated due to different ICT systems and busy schedules on both sides.

“We can land on the moon, but we still haven’t devised a system for effective communication. We’re still using fax machines, which is quite dramatic. The inability to consult each other’s systems is always a significant challenge.” S08, Male, 54 years, Medial Specialist, Internal medicine.

#### GP competences

The comprehensive care of a GP was considered to be an advantage as well as a disadvantage. Secondary care providers stated that the lack of specialization was associated with less experience with cancer-specific physical examinations and limited knowledge about treatment and long-term effects. This could limit GPs’ capacity to reassure patients according to some interviewees. At the same time, their experience with psychosocial care was seen as a strong benefit, as cancer is associated with distress and affects various spheres of life. The familiarity of the GP helps to better integrate patient’s context and makes personalized follow-up care possible.

“The GP has much more experience with people who are worried but not ill. We have way more experience with people who are truly diseased and in need of treatment. And by the time they come in for follow-up they are essentially no longer ill.” S03, Male, 64 years, Medical specialist, Surgery.

### Requirements for substitution of care

Requirements expressed by secondary care providers were thematized into four subthemes “Hospital organization,” “Innovations,” “GPs’ expertise,” and “Resources.”

To improve communication between primary and secondary care, they suggested appointing a case manager in the hospital, preferably a nurse, who can verify that all follow-up examinations have been performed, that no abnormalities have been missed, and that there is a clear delineation of responsibilities.

“It means that you are only doing duplicate work, resulting in a deterioration of the quality of care. It’s like having two captains on the same ship, both trying to steer in different directions, often at the expense of the patient.” S10, Male, 53 years, Medical specialist, Internal medicine.

Another suggestion was to involve a specialized nurse practitioner from the hospital to organize consultations in the GP practice, as they are already trained in this follow-up care. To improve accessibility for patients re-referred by their GP, most specialists agreed that secondary care should keep slots in their schedules to provide prompt consultations. In addition, especially surgeons and radiotherapists mentioned that clear radiological reports for GPs should be mandatory as GPs have no access to hospital data.

“The most important thing is that the patient feels safe, he/she must also be able to have confidence that the GP can handle it [the follow-up care] and that if there are any questions, GPs can easily and directly refer a patient back to the hospital.” S12, Female, 43 years, Medical specialist, Surgery.

All participants felt that the oncological expertise of the GP should first be increased before a formal role is considered. Sufficient case-load and training are mandatory to develop the appropriate knowledge and skills like performing physical examinations and interpreting test results.

“And education is important, you have to make clear what CEA stands for and what to do if the CEA is slightly increased or the CEA is way too high. The interpretation of an ultrasound is not really necessary, a report should be sufficient. We cannot interpret these ultrasound images in detail ourselves.” S17, Male, 41 years, Medical specialist, Surgery.

Most participants believed that expertise could be increased by developing clear, evidence-based protocols for primary care. In addition, the majority desired an update of ICT facilities with one national system for electronic patient files for both primary and secondary care. This system would enable patients to be automatically invited based on their follow-up schedule.

“Well, I think that it [oncological follow-up] would get lost in the everyday things, [in the hospital] we make clear follow-up calls for in a year and, yes, I don’t think a GP has such a system. You can say, the patient should keep an eye on these appointments, but then I don’t know if that will work out. So that people do not fall between two stools.” S01, Female, 59 years, Medical specialist, Internal medicine.

All participants believed that sufficient resources in primary care were necessary to provide such care, like an adequate number of staff members, time, sufficient remuneration, and physical workspace.

### Implementation strategy

After asking how the requirements mentioned should be organized/implemented in practice (although not everyone was enthusiastic), two subthemes were identified: patients’ specific characteristics (“Patient groups”) and “Effectuation.”

#### Patients group

Most secondary care providers differentiated between patients who are eligible for primary follow-up care and those who are eligible for secondary follow-up care. Some participants classified patients as eligible for primary care based on cancer-specific characteristics such as patients with a low recurrence risk or an uncomplicated first year after treatment. Others considered age to be a more discriminating factor and thought that frail older patients with comorbidities were better off in primary care. In addition, almost all internists agreed that young (breast) cancer patients with hereditary cancers should stay in secondary care. A few proposed selections should be based on patients’ wishes, with less focus on the disease itself, e.g., patients who are ready to resume daily life.

“We have to choose based on, well, what is most relevant for this patient. I can imagine that you follow T4M2 stage patients mainly with the internist and that you refer T1 and 0 stage carcinomas to the local specialists such as the GP.” S03, Male 64 years, Medical specialist, Surgery.

#### Effectuation

In case of more formal involvement of GPs in the future, most participants proposed a shared-care model. In particular, some internists were reluctant to transfer all oncological follow-ups to primary care due to the concerns mentioned above. The majority of the participants felt that it was particularly important that GPs are willing and confident enough to provide parts of the follow-up care. Participants agreed that an individual follow-up plan should be composed, taking into account the type of cancer, treatment history, and personal wishes of a patient. Hardly anyone dared to name a specific period after which GP-led follow-up was feasible (in a shared care setting), because this differs per patient. However, most surgeons and radiotherapists agreed that 1 year after finishing treatment, an evaluation should be organized with all doctors including the GP and the patient to discuss the future follow-up. Medical internists did not immediately see an active role for the GP at this time, but did see advantages in an evaluation (three-way-conversation) in which all healthcare providers and the patient were involved.

“I think that it is nice, both for the specialist and patient, to see each other again at least after the surgery. So I’d say, for example, do the first six months of follow-up and the first annual appointment in the hospital and thereafter transfer it to the GP, that we make such an agreement together.” S11, Male, 38 years, Medical specialist, Surgery.

In general, they proposed starting with patients with the most prevalent cancers like colorectal, breast, and prostate cancer. The quality of primary follow-up care should be at least at the level of secondary care. This should be monitored in the first years by predefined indicators.

“I think where the profit lies for us, are indeed the large patient groups, so the breast and colorectal carcinomas, and I think a lot of patients with prostate carcinomas are still followed in the hospital but may not belong there anymore. We could start with these groups.” S13, Female, 52 years, Nurse practitioner, Surgery.

## Discussion

### Summary

Secondary care providers currently involved in cancer follow-up care identified perceived barriers and facilitators to formal involvement of GP practices. Among the barriers were the perceived inability to perform structured follow-up in GP practices and risk of re-referral delays. Additionally, concerns about the lack of specialized oncological knowledge among GPs were voiced. At the same time, the accessibility, familiarity, and contextual knowledge of GPs were reported as clear benefits to formal GP involvement.

When asked about requirements for future formal involvement of GPs, many interviewees suggested improvements to hospital logistics to better accommodate shared care between hospital and primary care. Additionally, formal training for GPs, sufficient case-load, and proper staffing, remuneration, and time for GPs were identified as essential prerequisites for follow-up in primary care. Finally, they suggested that formal GP involvement should first be implemented for frail older patients and for prevalent cancer types, whereas patients with a high risk of recurrences or a complicated course were suggested to remain in hospital-based follow-up. Individualized follow-up plans were mentioned as a necessity.

### Comparison with literature

Our finding that specialists voiced concerns about GPs’ ability to provide structured follow-up was also reported by GPs in another study [17]. This is interesting because GP practices in the Netherlands are currently responsible for managing structured care for patients with chronic diseases like diabetes, respiratory problems, and cardiovascular risk management, and they perform this particularly well. This care is typically provided by trained nurse practitioners under GP supervision. One could argue that the fact that many cancer patients have chronic comorbidities and frequently visit their GPs [8, 26–28], integrating cancer follow-up into this type of care is a viable approach. However, it is plausible that specialists and GPs view cancer as different from the aforementioned chronic diseases, because of its complex pathophysiology and rapidly developing knowledge base. This could also be reflected in our finding that specialists felt that GPs lack specialized oncological knowledge. Earlier studies reported similar concerns voiced by GPs; they felt that their knowledge of follow-up routines, test interpretation, side effects, and long-term treatment effects for cancer was not up-to-date and they might lack the required experience [17, 29]. However, a qualitative study involving both primary and secondary care providers found that additional specific training for GPs was not deemed necessary in a shared care setting, with clear protocols and effective communication being considered more important [30].

Several studies have shown that cancer follow-up in primary care is feasible and safe, showing no differences in recurrence detection and survival [4, 6]. Yet, secondary care providers in our study expressed concerns about re-referral delays in case of a suspected recurrence, a challenge also highlighted in other studies [15]. Earlier studies showed that a significant proportion of recurrences are detected symptomatically by GPs in between follow-up visits [31]. In addition, meta-analyses comparing intensive and less-intensive follow-up for breast or colorectal cancer showed no differences in overall survival [32–34]. One might even hypothesize that regularly scheduled follow-up visits could also cause delays, either from the patient or the doctor, as both may wait for the next planned appointment.

A clear benefit of a GP involvement in follow-up care reported by specialists was the accessibility and familiarity of GPs, potentially reducing anxiety and stress around follow-up examinations and generally improving patient well-being. This finding is also supported by studies among GPs and patients, which highlight improved continuity of care, increased psychosocial attention, better accessibility, and greater familiarity [15, 17, 18, 35–37]. GPs generally feel well-equipped to provide psychosocial care in cancer follow-up, and with their long-term patient relationships and deep understanding of individual goals and contexts, they are well-equipped to further personalize follow-up care. This may explain why there are also studies showing that the majority of general practitioners felt proficient in performing cancer follow-up care tasks [38].

In addition, the COVID-19 pandemic has led to significant changes in clinical practice, with telehealth playing a crucial role in improving communication between primary and secondary care [39–41]. Communication issues remain a challenge, as does the current shortage of general practitioners, which hinders the formal involvement of GPs.

### Implications for practice

A shared-care model, leveraging the expertise of medical specialists and the accessibility of GPs, may prove to be an effective way to increase the GPs’ role in cancer follow-up. It can enhance the personalization, effectiveness, and efficiency of oncological follow-up. Rather than a standardized, disease-focused follow-up protocol, it would allow for care tailored to patient-specific goals and priorities. With the growing evidence supporting less intensive follow-up, this approach appears feasible. According to medical specialists as well as GPs and patients [17, 18], key components include the individualized care with input from specialists, GPs, and patients, as well as clear re-referral pathways and communication between care providers. Overcoming communication barriers and addressing GPs’ lack of confidence through training and rapid specialist consultations are also crucial.

In practice, using colorectal cancer as an example, shared care could potentially begin immediately after treatment to ease the transition, with the frequency and intensity of follow-up tailored to the patient's needs, preferences, and recurrence risk. An individualized follow-up plan should be developed, ideally through a three-way discussion, although this may not always be feasible. A visit to the GP early in the follow-up period should be scheduled to re-establish contact and discuss patient preferences. For instance, the first year might include alternating appointments, such as GP visits at 3 and 9 months and specialist follow-ups at 6 and 12 months, depending on the agreed-upon plan. The recent SCORE RCT showed that this follow-up schedule with shared care was non-inferior to current hospital care and acceptable to patients, with no differences reported in quality of life or recurrence detection [11, 42]. GPs could manage CEA monitoring, perform physical examinations as necessary, and assess the patient’s well-being. After the colonoscopy or CT scan at 1 year, GP care could potentially expand, depending on the patient’s risk profile and preferences, as suggested in this study. In such a shared-care model, patients, GPs, and specialists each have their roles. An individualized follow-up care plan can help patients take a more active role in their follow-up, for instance, by enabling them to schedule appointments themselves and take greater ownership of their care. To ensure continuity of care, however, it may be beneficial to have a hospital case manager or dedicated point of contact in primary care to coordinate and organize the alternating appointments between the GP and the hospital. Introducing a new care model requires time for adaptation; therefore, it is crucial to prepare patients early and clearly communicate the transition, so they eventually perceive it as the new standard of care.

### Strengths and limitations

This is the first study to explicitly examine the opinions of secondary care providers, on the formal involvement of the GP and, in particular on possible implementation strategies. We therefore consider this study a relevant addition to the discussion about possible future oncological follow-up strategies of care.

A possible limitation is that the interviews in this study concerned the views of secondary care providers based on their experiences with care in hospitals. There may be a lack of knowledge among them about the current working methods in general practice.

Moreover, adopting a deductive approach with predefined categories could have restricted the flexibility of our analysis, leading us to potentially overlook relevant information that did not conform to these categories. Nevertheless, we believe that while deductive coding may have somewhat limited our analysis, its integration with inductive coding has allowed us to delve deeper into these predefined topics. We think this approach effectively addresses our specific research question and ensures that our findings extend beyond simply reiterating existing information.

Lastly, in this study, we focused exclusively on breast cancer and colorectal cancer, although other types of cancer would also be of interest. We examined two of the five most common cancer types; however, while prostate cancer was a strong candidate, we also considered types with somewhat more complex follow-ups.

## Conclusion

The interviewed Dutch secondary care providers generally support more formal involvement of primary care in breast and colorectal cancer follow-up. A well-organized shared-care model with defined roles and clear coordination between secondary and primary care, and supported by individual patients, is seen as conditional. To involve GPs in cancer follow-up care, hospitals may need to adapt logistics, and GPs require formal training, resources, and time.

## Supplementary Information

Below is the link to the electronic supplementary material.

Supplementary file1 (DOCX 18 KB)

## Data Availability

No datasets were generated or analysed during the current study.
