# Supplementary material for: Organizing substitution of oncological follow-up to primary care: perspectives from secondary care providers
Source: J Cancer Surviv. 2025 Mar 6;20(4):1648–57. doi: 10.1007/s11764-025-01764-x (PMC13375917; doi:10.1007/s11764-025-01764-x)
Supplement: Supplementary file 1 — Supplementary file1 (DOCX 18 KB) [file 11764_2025_1764_MOESM1_ESM.docx]

**Organizing substitution of oncological follow-up to primary care: perspectives from secondary care providers**

Geertje B. Liemburg^1^, Joke C. Korevaar^2,3^, Annette J. Berendsen^1^, Marjolein Y. Berger^1^, Daan Brandenbarg^1^

1. University of Groningen, University Medical Center Groningen, Department of Primary and Long-term Care, P.O. Box 196, 9700 AD Groningen, the Netherlands
2. NIVEL Netherlands Institute for Health Services Research, Utrecht, the Netherlands
3. The Hague University of Applied Sciences, Faculty of Health, Nutrition and Sport, The Hague

**Corresponding author:** Geertje Liemburg, [g.b.liemburg@umcg.nl](mailto:g.b.liemburg@umcg.nl)

**Online Resource 1: Study reported according to COREQ Criteria**

Items of the COREQ checklist not specified in the manuscript are reported below.
**Domain 1: Research team and reflexivity**

- Credentials. What were the researcher’s credentials? (e.g., PhD, MD)
- Occupation. What was their occupation at the time of the study?
  GBL: MD, GP and PhD-student; JCK: PhD, epidemiologist; AJB: MD, PhD, GP, epidemiologist; MYB: MD, PhD, Professor of General Practice; DB: PhD, epidemiologist.
- Gender. Was the researcher male or female?

GBL, JCK, AJB, MYB: female; DB: male. JCK, MYB, AJB did not participate in data gathering.

- Experience and training. What experience or training did the researcher have?

DB is an experienced interviewer and focus group leader. GBL received training in qualitative methods (advanced courses) and was trained in performing interviews by DB.

- Relationship established. Was a relationship established prior to study commencement?

There was no relationship prior to the study between the participants and researchers.

- Participant knowledge of the interviewer. What did the participants know about the researcher? (e.g., personal goals, reasons for doing the research)

Prior to the study, all participants received an invitation letter with background information about the study and study goals. At the beginning of an individual interview, the researchers introduced themselves, their backgrounds, and their roles. Also, all participants were invited to tell something about themselves and their reason to participate. They were also encouraged to ask questions.

**Domain 2: Study design**

- Presence of non-participants. Was anyone else present besides the participants and researchers?
  No one beyond participants and researchers were present during the interviews.
- Field notes. Were field notes made during and/or after the interview or focus group?
  Yes, field notes were made by the researcher after the individual interviews.
